# Supporting Smart Home Scenarios Using OWL and SWRL Rules

**DOI:** 10.3390/s22114131

**Published:** 2022-05-29

**Authors:** Roberto Reda, Antonella Carbonaro, Victor de Boer, Ronald Siebes, Roderick van der Weerdt, Barry Nouwt, Laura Daniele

**Affiliations:** 1 Department of Computer Science and Engineering, University of Bologna, 40136 Bologna, Italy; roberto.reda@unibo.it; 2 Computer Science Department, Faculty of Science, Vrije Universiteit Amsterdam, 1081 Amsterdam, The Netherlands; v.de.boer@vu.nl (V.d.B.); r.m.siebes@vu.nl (R.S.); r.p.vander.weerdt@vu.nl (R.v.d.W.); 3 TNO—Netherlands Organization for Applied Scientific Research, 2597 The Hague, The Netherlands; barry.nouwt@tno.nl (B.N.); laura.daniele@tno.nl (L.D.)

**Keywords:** IoT, home automation, smart home, SAREF, ontology, SWRL, cognitive systems

## Abstract

Despite the pervasiveness of IoT domotic devices in the home automation landscape, their potential is still quite under-exploited due to the high heterogeneity and the scarce expressivity of the most commonly adopted scenario programming paradigms. The aim of this study is to show that Semantic Web technologies constitute a viable solution to tackle not only the interoperability issues, but also the overall programming complexity of modern IoT home automation scenarios. For this purpose, we developed a knowledge-based home automation system in which scenarios are the result of logical inferences over the IoT sensors data combined with formalised knowledge. In particular, we describe how the SWRL language can be employed to overcome the limitations of the well-known trigger-action paradigm. Through various experiments in three distinct scenarios, we demonstrated the feasibility of the proposed approach and its applicability in a standardised and validated context such as SAREF

## 1. Introduction

Over the last few years, the home automation sector has seen significant adoption of IoT devices performing the most various sensing and actuating capabilities for a plethora of different domotic tasks. Basically, IoT domotic devices are size-contained wireless systems which can be accessed and programmed through the internet to monitor or control home attributes such as air conditioning and heating, lighting, surveillance and home appliances.

IoT home automation, besides increasing entertainment and user comfort, can provide several potential benefits in other crucial areas such as home safety [1], energy efficiency and preservation [2], and elderly care [3]. However, current home automation systems do not fully exploit the intrinsic potential of IoT devices due to two critical challenges they face: the high heterogeneity of the devices and protocols which results in limited interoperability (a common issue that plague the IoT landscape in general); and the limited expressivity of the paradigms adopted to program domotic scenarios [4]. In fact, the majority of commercially available IoT programming environments are proprietary technologies based essentially on the low level abstraction trigger-action model which is limited to processing only a small number of different input data sources (normally, only sensor data gathered by devices from the same vendor) and lacks reasoning features.

For example, a typical current domotic scenario for room temperature regulation consists of turning on the air conditioner set to the desired temperature and letting the internal thermostat do the rest. However, a smart IoT system could be embedded with the knowledge to make it be able to autonomously find the best way to cool down the room temperature. For instance, by combining data from internal thermometers and online weather services it can decide to just open the window and let the fresh air in instead of simply turning on the air conditioner thus saving energy. Additionally, the same system could automatically close the window if it starts raining outside or when the residents leave the house thus guaranteeing safety.

Achieving such complex behaviour requires the smart home system to be able to combine and analyse data coming from heterogeneous sources according to some formalised knowledge [5,6]. Consequently, automated reasoning capabilities and the adoption of a higher abstraction programming paradigm becomes essential as well [7].

Semantic web (SW) technologies consist of a set of open recommendations for associating data to their formal meaning and have been shown to constitute an appropriate means for achieving data interoperability in IoT systems [8,9]. Additionally, SW naturally enable inference capabilities over semantically annotated data that cannot be obtained using other traditional programming languages. Numerous studies in the literature have shown that SW technologies can be a suitable approach to tackle the complexity of many specific tasks in specific areas of modern IoT home automation [10,11,12].

The use of SW technologies, in particular OWL ontologies, as a means to overcome the poor interoperability in the IoT field has been recently largely investigated [5,13,14]. One notable example in the smart home field is the Smart Appliance REFerence ontology (SAREF) [15]. SAREF is an OWL-DL ontology, created in close interaction with the industry, that aims at formally describing the core concepts in the smart appliances domain. The ontology defines concepts for modelling the devices, their tasks and the functions they perform to accomplish the tasks. It also enables the description of the device energy profile and power profile. The ontology is designed to be easily extendable and can be used as basis for creating more specialised ontologies such as SAREF4Health, an extension for IoT-based healthcare systems [16]. Moreover, SAREF has been standardised by the European Telecommunications Standards Institute (ETSI) and experimentally validated [17].

Rule-based programming approaches such as the trigger-action paradigm are widely employed in actual IoT home automation systems for defining domotic scenarios due to their simplicity and intuitive use [18]. For example, IFTTT is one of the most popular tool for programming IoT scenarios using trigger-action rules [19]. However, even though IFTTT partially extends the expressivity of the simple trigger-action model through the integration of web services, it suffers from several limitations such as a low-level abstraction and low generalisation due to the lack of actual semantics support and reasoning capabilities [4,7]. Indeed, besides providing semantics to IoT data, SW technologies also provide support for dealing with the collected information, that is they naturally enable reasoning capabilities over the semantically annotated data, especially by means of rule languages [20].

In [21], Corno et al. employed SW technologies to overcome the low level abstraction of IFTTT rules in end-user development (EUD) environments. The authors created EUPont, a high-level OWL ontology that provides abstract representations for EUD programming environments for the IoT.

The applicability of SW ontologies and rule languages in home automation has been investigated by Bonino et al. [22]. In their work, the authors employed an OWL ontology (i.e., DogOnt ontology [23]) to provide a common semantics and features description for the devices involved, and two rule languages (i.e., SWRL and Jena rules) to perform reasoning. In their system, rules are defined to evaluate structural and state properties of the home environment.

Fensel et al. in [24] describe the SESAME-S project (SEmantic SmArt Metering Services for Energy Efficient Houses) which makes use of linked data to assist home tenants in making informed decisions and controlling home energy consumption. Owl ontologies are used to semantically annotate data regarding automation devices, consuming measurement and energy pricing. Rules are employed to implement policy-based decision-making mechanisms.

More recently, in [25] Saba et al. have proposed a system for energy management in smart home environments. The OWL ontology on which the system builds up provides a formal representation of the energy aspects of the appliances and other domotic environment elements such as the extent by which they positively or negatively influence the consumption of electrical energy. SWRL rules are used as reasoning mechanism to implement energy saving scenarios without compromising tenants’ comfort.

Previous works in the domotics field that have adopted SW technologies focus only on specific home automation tasks (e.g., energy management). Accordingly, automated reasoning through OWL and SWRL is performed over solely ad hoc written ontologies.

Indeed, the necessity of an ontology-agnostic approach in SW solutions have been clearly highlighted in [26] for building-level automation systems. In this work, the authors propose BRICK (Building’s Reasoning for Intelligent Control Knowledge-based System) as an integrated system for intelligent building energy and security management.

Stemming from these results, the aim of our work is to suggest a unifying approach in home automation systems by demonstrating how the Semantic Web Rule Language (SWRL) can be employed as a general purpose programming paradigm to implement advanced domotic scenarios which are not limited to work only in a specific domotic area or rely only on ad hoc ontologies. For this purpose, we developed the Semantic Smart Home System (SSHS), a knowledge-based system which is capable of performing home automation facilities by executing SW rules over in situ collected IoT sensor data potentially combined with external information sources (e.g., curated ontologies or third party web services). Our work builds upon the existing standardised ETSI Smart Applications REFerence (SAREF) ontology (the SAREF ontology is available at https://saref.etsi.org/ (accessed on 30 March 2022)), which, as mentioned above is specifically designed to enable semantic interoperability in IoT systems including domotics [15,17]. It is important to note that SAREF is a general purpose ontology in the IoT domain, it has not been developed for a single specific project, therefore it cannot be considered an ad hoc solution.

The experiments that we have carried out showed the feasibility of our approach and the efficiency of the SSHS in realistic real-life settings.

The remainder of this paper is organised as follows. In Section 2, a detailed description of the architecture and the functioning of the SSHS is provided and illustrates how the SWRL language can be used as a home automation scenario programming paradigm. The experiments that have been carried out using the proposed approach are described in Section 3. Finally, in Section 4 concluding remarks and future research directions close the paper.

## 2. Materials and Methods

The main objective of this study is to demonstrate that SW technologies can be employed to realise home automation scenarios that can fully exploit the potential offered by modern IoT devices. Indeed, in order to achieve an overall higher level of automation, the heterogeneous amount of information collected by IoT devices crucially requires to be integrated and analysed accordingly to some sort of formalised knowledge, such as OWL ontologies.

To achieve this goal we developed the SSHS which is a knowledge-based framework that can execute complex home automation scenarios. In SSHS, automatic actions are the result of SWRL rules executed over semantically annotated IoT data potentially combined with external information sources such as curated ontologies or web services. In SSHS, semantics also provides the expressiveness and abstractions which facilitates the task of tackling the programming complexity. That is, in SSHS, rules are expressed in terms of high level entities rather than the respective lower level of the sensor raw values or device specific actuator commands.

In our study, the SSHS operates within a SAREF-based environment. A SAREF environment, in this context, is essentially composed of two elements: a set of knowledge bases and a knowledge engine. The term knowledge base refers to a general entity which can communicate and exchange data semantically annotated according to the SAREF ontology or one of its extensions. For instance, any IoT device, such as a door switch or a thermometer, in a SAREF environment can be seen as a knowledge base. All the knowledge bases are connected to the knowledge engine through a smart connector (i.e., a generic component that is being developed by the InterConnect consortium (InterConnect consortium: https://interconnectproject.eu/consortium/ (accessed on 30 March 2022))). Knowledge bases configure their smart connector with their specific capabilities and RDF graph patterns. The knowledge engine uses these to function as the coordinator system that allows the knowledge bases to exchange information to each other. The RDF graph patterns are used by the knowledge engine to route the data. Further information about the Knowledge Engine can be found in the InterConnect public git repository (InterConnect public git repository: https://gitlab.inesctec.pt/interconnect-public/knowledge-engine/ (accessed on 30 March 2022)) and deliverable 5.2 (InterConnect Deliverable 5.2: https://interconnectproject.eu/wp-content/uploads/2021/02/InterConnect_WP5_D5.2-DataFlowManagement_draftVersion.pdf (accessed on 30 March 2022)).

**Figure 1:**
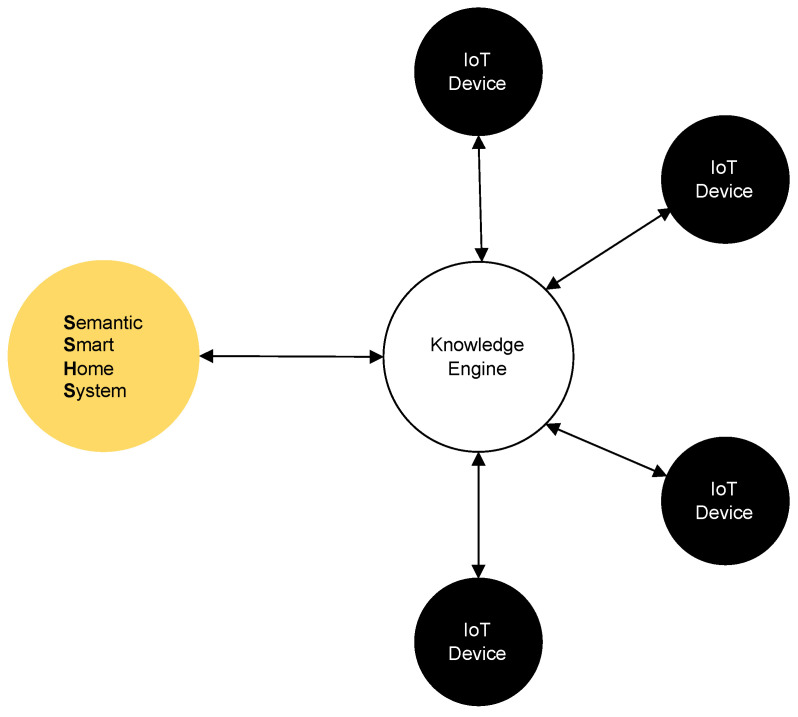
The schema depicts an example of SAREF environment setup, as intended in this study, composed of four IoT devices that exchange data in RDF format, through the knowledge engine, with the Semantic Smart Home System.

The SSHS we developed can be seen as a SAREF knowledge base that once connected to the knowledge engine periodically receives sensor data gathered from the IoT devices, executes semantic rules over them, and sends commands back to control the actuators. Since the SAREF ontology provides a common semantics, rules can be written device vendor independently. An example of an SAREF setup is shown in Figure 1, in which four IoT devices collect data from the environment and exchange them with the SSHS that can eventually send commands back to them.

**Figure 2:**
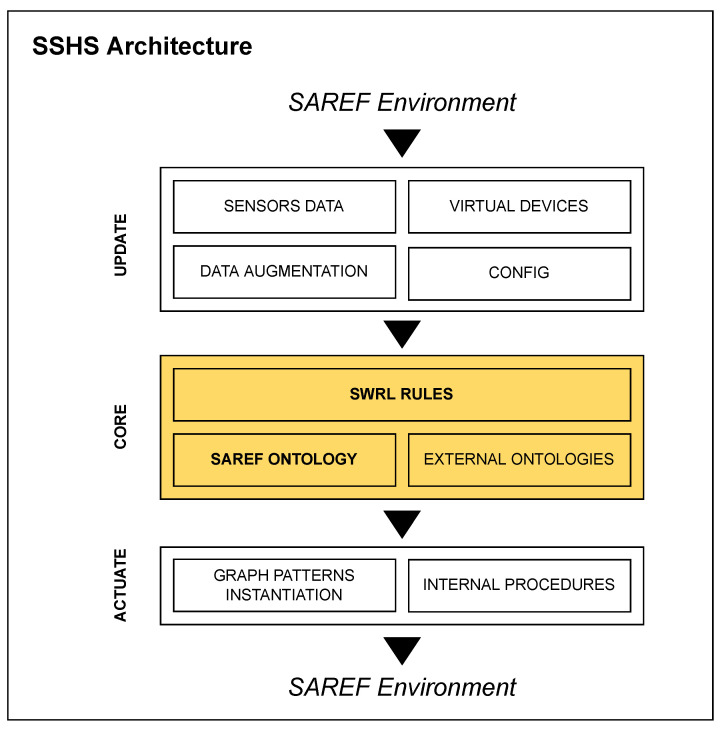
The Semantic Smart Home System architecture. The system is made up of three main components: the *update component* which collects data from the SAREF environment and constructs the RDF graph representing the current house status, the *core component* in that executes the SWRL rules over the content of the main graph, and the *actuate component* which inspects the reasoning result and sends commands back to the actuators. These tasks are executed sequentially in a loop.

Figure 2 depicts the main architecture of the SSHS. The SSHS is made up of three main components: the update module, the core system and the actuate module. These components realise three phases which are executed in order and cyclically.

The first phase is the update phase which is performed by the update module. During the update phase, the system gathers all the sensor data measured at that moment by the IoT devices installed in the house. Optionally, the incoming data can be arbitrarily augmented through a software procedure. For example, if a physical device does not provide in its output a timestamp, it can be added at this stage. Data from virtual devices (i.e., web services or external data sources) and configuration information are collected as well. The output of the update phase is an RDF graph which represents the current status of the house combined with all the other information gathered.

The second phase is the rules execution phase that is performed by the core system. The core component represents the most important part of the SSHS. It executes the semantic web rules over the RDF graph produced during the previous phase. The semantic web rules are expressed using the SWRL language. The SAREF ontology and optionally other external OWL ontologies, takes part in the inference process as well. In our SSHS implementation, the OWL classification and the rule execution are performed by the reasoner Pellet which is an open source OWL-DL reasoner that also features an embedded SWRL inference engine [27].

The third and the last phase is the actuate phase that is performed by the actuate module. The actuate module first analyses the result of the reasoning process and then accordingly it generates the graph pattern instantiations (i.e., the device commands) to be sent to the knowledge engine in order to operate the actuators such as turning on a lamp or closing a shutter. Along with graph pattern instantiations, during the actuate phase internal procedures are executed to modify the status of the virtual devices.

### 2.1. IoT as Data Sources

Reasoning over IoT sensor data combined with external information sources is the key feature of the SSHS. Within the SSHS, every data source can be modelled as an IoT device. In SSHS an IoT device can be of three kinds: physical device, augmented device and virtual device.

Physical devices correspond to the actual IoT devices materially installed inside the house. For instance, a physical device could be a door switch sensor mounted on a door frame or a thermometer which gauges the room temperature. For each physical sensor in the house, the update module collects its status information as an RDF graph as it comes as output from the device itself.

An augmented device is basically a physical device to whose output graph one or more pieces of extra information are added through a software procedure during the update phase. This is necessary because unfortunately IoT devices do not always provide by default all the essential information that might be needed to write rules, such as their status history. For instance, most of the colour changing light bulbs available on the market provide in their status only the current colour which is actually being displayed while sometimes it could be necessary to keep track of the previous configurations to restore a previous state after temporarily changing it.

A virtual device is a generic IoT device whose functionalities are fully software emulated. Virtual devices do not have a corresponding physical device installed in the house. For example, the information that an IoT weather station provides (e.g., temperature, humidity, wind speed, rainfall and solar radiation) can be also obtained from an online weather web service thus making unnecessary (at least in some cases) the presence of an actual physical device in the house. Virtual devices can also be used to provide functionalities that are not natively available in SWRL or OWL run-time environments, such as functions to retrieve the current date. However, in SSHS, a clock can be easily implemented as a virtual IoT that provides as output the actual date time. Virtual devices can either be implemented as software procedures invoked by the update module or as separate SAREF knowledge bases connected directly to the knowledge engine.

### 2.2. Implementation Details

We implemented a prototype of the SSHS using the Python language. The Owlready2 [28] framework was used to connect the update and the actuate component to the core component which is enabled by the Pellet reasoner [27]. Pellet is an open source OWL2-DL reasoner that natively supports SWRL rules execution.

Some demo code is available at https://github.com/robertoReda/sshs (accessed on 30 March 2022).

### 2.3. Rules Writing and Scenarios

A home automation scenario refers to a set of actions that are performed when certain conditions are met. Most of the current home automation tools adopt the trigger-action model as a home automation scenario programming paradigm [4]. In trigger-action systems the desired behaviour is specified by means of rules in the form “if-then” where the “if” part of the rule checks whether a particular event (i.e., the trigger) has occurred and the “then” part specifies the action that should be executed in response. For instance, a typical scenario rule could be: “If the leak sensor detects some water, then turn off the washing machine”. Rule-based languages provide an intuitive way to program home automation scenarios especially when IoT devices are involved [4]. However, IoT home automation tools that do not adopt semantics suffer from several important limitations. First, the impossibility to define generic sets of rules for devices which have similar functionalities instead of vendor specific rules. Second, triggers and actions can be specified only in terms of device output values and device commands which implies a low level of abstraction. Finally, actions are determined on the sole base of data input sources due to the lack of reasoning capabilities.

The Semantic Web Rule Language (SWRL) [29,30] allows the definition of rules in terms of OWL entities, that is, it combines the ease of rule-languages with the capability to perform automated reasoning. SWRL is the means by which domotic scenarios are programmed in the SSHS. In SSHS automatic actions are determined by analysing the IoT input data according to knowledge expressed in OWL ontologies or in the rules themselves. In other words, in SSHS automatic actions are the result of logical inferences drawn over the RDF graph that represents the current status of the house.

Technically, SWRL provides a high-level abstract syntax for horn-like rules fully compliant with OWL semantics. A SWRL rule is composed of two parts, an antecedent and a consequent where both the antecedent and the consequent consist of a positive conjunction of atoms. Since SWRL rules are expressed in terms of OWL concepts, atoms can be either individuals, properties or classes defined within the knowledge base. This feature of SWRL is particularly important in SSHS because, in rule definition, IoT sensor data and knowledge can be composed together in a seamless way, thus allowing to achieve a higher level of abstraction. For example, a rule to automatically turn on a lamp when the natural light drops could be written as shown in Listing 1 regardless of how the low light condition of the room has been actually determined. For instance, it could be either obtained by analysing the output of a physical photocell (this would be the only possible way with a trigger-action system) or inferred using the information contained in an ontology given the period of the year and the current time. Alternatively, the same information could be achieved by consulting a web service.

Listing 1 **A SWRL rule tha can be used to switch on a lamp in case of low light condition inside a room.**

lowLightLevel(Room) ⇒ switchOn(Lamp)

This approach overcomes the limitations of the simple trigger-action model; the higher level of abstraction in rule definition helps significantly to tackle the complexity of defining advanced home automation scenarios. Most importantly, automated reasoning capabilities provided by OWL and SWRL dramatically increase the overall degree of automation that can be potentially achieved by the system.

In trigger-action programming, to check whether scenario conditions are met two types of triggers can be employed: event triggers and state triggers [31]. Event triggers refer to when an asynchronous event occurs, that is when a certain condition becomes true at a particular instant in time. For example, when a button is pressed or when a presence sensor detects a person entering a room. State triggers occur when a condition is true over a period of time. For instance, the condition that it is raining outside or the temperature is above a certain threshold.

In the SSHS, the only way to determine where scenario conditions are met is to analyse the current status of the house represented by the RDF graph that is constructed during the update phase. Since the home status RDF graph is sampled at regular intervals, state triggers are naturally supported, but asynchronous events can not activate any immediate response. However, in SSHS, event triggers can be easily simulated by checking whether the event condition has occurred in the near instant using the timestamp provided by the IoT devices. For example, the rule in Listing 2 can be used to automatically turn on the garden lights when a car passing through the gate is detected by a photocell.

Listing 2 **This SWRL rule switches on the garden lights if the photocell has detected the passage of a car within the last ten seconds.**

hasClosedState(photocell)∧ hasTimeStamp(photocell, ?ts)∧ currentTime(?ct)∧ swrlb:subtract(?ct, ?ts, ?delta)∧ swrlb:greaterThan(?delta, 0)∧ swrlb:lessThan(?delta, 10,000)⇒ switchOn(gardenLight)

The above rule checks whether the difference between the current time and the instant reported in the time stamp is below a certain threshold. The rule can be simplified by defining another rule which generalises this concept as shown in Listing 3.

Listing 3 **The first rule is used to detect that a generic event has happened within the last ten seconds while the second rule makes use of the first one to control the garden lights.**

hasClosedState(photocell)∧ currentTime(?ct)∧ swrlb:subtract(?ct, ?ts, ?delta)∧ swrlb:greaterThan(?delta, 0)∧ swrlb:lessThan(?delta, 10,000)⇒ tenSecondsEvent(?device) tenSecondsEvent(gardenLight) ⇒ switchOn(gardenLight)

Generally, time stamps enable time reasoning capabilities that are particularly useful for dealing with ordered events. For example, an action is performed only if a person has pressed a button after entering a room and not vice versa.

It is worth to note that neither SWRL nor OWL natively provide current time functionality which is essential to simulate event triggers in the SSHS. However, time information can be easily introduced into the system by using virtual IoT devices (i.e., in this case a simulated clock device).

Similarly to triggers, actions can be distinguished into instant actions, sustained actions, and extended actions [31]. Instant actions occur at a particular instant of time such as “turn on the light”. Sustained actions are performed as long as a condition holds, for example, “light is on as long as there is someone in the room”. Extended actions are performed for a specific time interval, therefore time stamps are needed to accomplish this task. For example, “change the light colour to red for 10 s”.

After a sustained or extended action it is often necessary to resume the previous state of the device. Since it is not possible to store indefinitely new knowledge within the knowledge base (i.e., the house state graph is rebuilt at each update phase), augmented IoT devices can be employed when the devices do not natively provide a status history or when it is not possible to infer the previous state from the current graph.

OWL and consequently SWRL knowledge bases are monotonic; new knowledge can be added, but existing knowledge can not be retracted or modified. In the SSHS, rules can not directly change the state of the IoT devices. For example, if a lamp has to be turned on, rules classify the respective OWL individual into a desired state which represents the action that should be taken. Only during the update phase actual commands are sent to devices according to the classification results in order to reflect changes. Actual changes to the environment are visible within the knowledge base only after the next update phase has been completed.

## 3. Results

An IoT home automation system is essentially composed of a variable number of devices that include both sensors to perceive the domestic environment and actuators to perform actions on it, connected to a programmable unit that implements the control logic (i.e., the domotic scenarios). The majority of traditional IoT home automation systems adopt the trigger-action model as a programming paradigm for defining scenarios, according to which every time certain conditions are met, a specific action is executed.

The SSHS that we developed extends the trigger-action model by introducing automated reasoning capabilities in the process. In our system, domotic scenarios are expressed by means of SWRL rules that combine knowledge with trigger-action definitions. Therefore, the automatic actions are not only determined based on sensor data, but are the result of logical inferences enabled by the underlying semantics provided by the SAREF ontology. The aim of this approach is to achieve greater expressivity and a higher level of abstraction needed to build knowledge-enabled and reasoning-capable home automation systems, so that the potential offered by IoT devices can be fully exploited.

In order to show the feasibility of the proposed approach, we have designed and tested on our prototype several different knowledge-involving domotic scenarios that operate under different conditions with different kinds of IoT devices. These use cases also are meant to demonstrate that scenarios in SSHS can be based on web standards and public ontologies and implement well-defined reasoning without the necessity of ad hoc control programs or even ad hoc ontologies.

Moreover, we evaluated the system performance by measuring the processing time taken by the reasoner to evaluate rules with different numbers of devices involved in order to demonstrate the efficiency of the SSHS in realistic settings.

Experiments were conducted using a simulated house environment. IoT devices output data were synthetically generated using a Python script. This testing method is particularly convenient since it is economical, significantly speeds up the process of recreating the desired experimental conditions and simplifies the analysis of the resulting system behaviour. Moreover, it is worth to note that since the semantics provided by the SAREF ontology makes data device-independent, synthetic generated IoT output graphs do not lack any relevant information that might be acquired in a real life setting.

**Figure 3:**
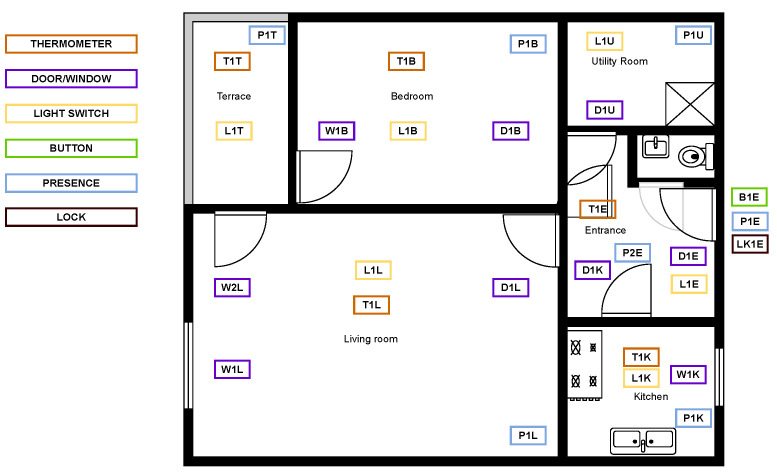
The schema represents the floor-plan of typical domestic environment equipped with various domotic IoT devices.

An example of a simulated domestic environment that we used as a testbed to carry out experiments is shown in Figure 3. In this case, the environment consists of a 6 room flat plus a terrace, equipped with 29 IoT devices including light switches, door/windows switches, thermometers and a presence detection sensor. An excerpt of the corresponding RDF graph model of the house is shown in Listing 4. As it can be seen, rooms are modelled as OWL individuals that belong to specific classes according to their functions within the house. SAREF4BLDG (SAREF4BLDG ontology: https://saref.etsi.org/saref4bldg/ (accessed on 30 March 2022)), an extension of the SAREF core ontology, has been employed for this purpose. For example, the terrace is represented by means of an OWL individual that belongs to the class s4bldg:BuildingSpace to indicate that it is an outdoor ambient.

Static knowledge about the environment and any other relevant information that cannot be inferred from the IoT data sources, such as the aforementioned spatial features of the house, can be introduced into the SSHS as configuration data in RDF format. There are no constraints on the quantity and the type of information that can vary significantly and strictly depends on the specific scenarios that make use of it.

Listing 4 **An excerpt of the RDF graph that represents the spatial features of the domestic environment shown in Figure 3.**

ex:Building rdf:type s4bldg:Building .ex : Bui lding s4bldg : hasSpace ex :House .ex:Building s4bldg:hasSpace ex:House .ex:House rdf:type s4bldg:BuildingSpace .ex:House rdf:type saref:FeatureOfInterest .ex:House s4bldg:hasSpace ex:LivingRoom .ex:LivingRoom rdf:type s4bldg:BuildingSpace .ex:LivingRoom rdf:type saref:FeatureOfInterest .ex:House s4bldg:hasSpace ex:Terrace .ex:Terrace rdf:type s4bldg:BuildingSpace .ex:Terrace rdf:type saref:FeatureOfInterest . ex:LivingRoom s4bldg:contains ex:Door .ex:Door rdf:type s4bldg:BuildingObject .ex:LivingRoom s4bldg:contains ex:Window .ex:Window rdf:type s4bldg:BuildingObject .s4bldg:BuildingObject rdfs:subClassOf s4bldg:PhysicalObject . ex:LivingRoom s4bldg:contains ex:LightSwitch .ex:LightSwitch rdf:type saref:Device .saref:Device rdfs:subClassOf s4bldg:PhysicalObject .ex:LightSwitch rdf:type saref:Actuator . ex:LivingRoom s4bldg:contains ex:Thermometer .ex:Thermometer rdf:type saref:Device .ex:Thermometer rdf:type saref:TemperatureSensor . ex:LivingRoom s4bldg:contains ex:PresenceDetector .ex:PresenceDetector rdf:type saref:Device .ex:PresenceDetector rdf:type saref:Sensor .

An example of an RDF graph representing an IoT data source and its associated measurement is shown in Listing 5. The graph regards a thermometer that is physically located on the terrace. The measurement value is reported along with the unit and the timestamp of the gauging instant in ISO-8601 format and Unix time.

Listing 5 **An excerpt of RDF graph that represents the output of an IoT thermometer annotated according to the SAREF ontology. The graph contains information about the physical collocation of the device within the house as well as the measurement in degree Celsius and the timestamp.**

ex:T1T rdf:type saref:TemperatureSensor ; saref:makesMeasurement ex:T1T_m1 ; s4bldg:isContainedIn ex:Terrace . ex:T1T_m1 rdf:type saref:Measurement ; saref:hasValue ‘‘25.5’’^^xsd:float ; saref:isMeasuredIn om:degree_Celsius; saref:hasTimestamp‘‘2020-12-02T14:30:00’’^^xsd:dateTime; smart:hasUnixTimestamp ‘‘1606919400’’^^xsd:integer .

During normal operation, the SSHS cyclically aggregates the various IoT data sources to construct an RDF graph that represents the current status of the domestic environment. Rules are then executed over this graph and the inferred knowledge is added back to it. Lastly, the system inspects the resulting graph to send, if necessary, commands to the actuator devices. For our tests, the last phase was omitted and the resulting graph was directly analysed using the Protege tool.

### 3.1. Energy Conservation Monitoring Scenario

Often, homeowners are unaware of the costs of some domestic energy wasting behaviours they involuntarily adopt, such as forgetting the windows open or leaving them open for too long a period of time. Therefore, monitoring in-home energy wasting in order to provide tenants immediate feedback can be a crucial feature for actuating an efficient energy wasting reduction in an IoT home automation system [32].

We implemented a domotic scenario that assesses the energy wasting level of a room according to the state of the windows (i.e., either open or closed). Top-down proceeding, the monitoring system consists of two main rules, shown in Listing 6, that classify the energy wasting level into two states: GreenEnergyState if there is no energy wasting (i.e., windows in the room are closed) or RedEnergyState if there is energy wasting (i.e., windows in the room are open).

Listing 6 **SWRL rules for classifying the energy wasting level in a room.**

smarthouse:ClosedWindow(?window)∧ s4bldg:isContainedIn(?window, ?room)⇒ smarthouse:GreenEnergyState(?room) smarthouse:OpenWindow(?window)∧ s4bldg:isContainedIn(?window, ?room)⇒ smarthouse:RedEnergyState(?room)

For each window of the house, first its state is detected. Then, the location of the window within the house is retrieved, and the corresponding room is finally classified accordingly.

A possible way to detect whether a window is open or closed could be achieved by installing a door switch IoT sensor on the window frame. For example, the closed window and the open window classification can be determined by retrieving the device state using the rules in Listing 7.

Listing 7 **The SWRL rules are used to detect whether a window is open using a switch sensor state.**

smart:Window(?window)∧ saref:hasState(?window, ?state)∧ saref:CloseState(?state)⇒ smarthouse:ClosedWindow(?window) smart:Window(?window)∧ saref:hasState(?window, ?state)∧ saref:OpenState(?state)⇒ smarthouse:OpenWindow(?window)

Since it cannot be inferred that a sensor is either installed on a window or a door, it is necessary to explicitly specify it as configuration data. This can be done either through the device settings or using rules. For example, the rule in Listing 8 states that the OWL individual W1L is a door switch installed on a window.

Listing 8 **The SWRL rule asserts that a DoorSwitch sensor W1L is installed on a window.**

saref:DoorSwitch(W1L) ⇒ Window(W1L)

The location of the window can be inferred through the location of the sensor (if it has been indicated in the device settings).

However, not always an open window causes heat loss, for example when the difference between the indoor and outdoor temperature is negligible. Therefore, to achieve more precision, rules can be modify so that the temperature is taken into account as in Listing 9.

Listing 9 **SWRL rules for classifying the energy wasting level in a room by taking into account several factors.**

smarthouse:OpenWindow(?window)∧ s4bldg:isContainedIn(?window, ?room)∧ smarthouse:hasIntExtTempDifference(?room, delta)∧ swrlb:greaterThan(?delta, 0.5)⇒ smarthouse:RedEnergyState(?room)

The internal temperature can be acquired by installing a thermometer inside the room, while the external temperature can be acquired by either installing a thermometer outside (e.g., on the terrace) or using an IoT virtual device that wraps an online weather web service. When the two measurements are available the difference can be easily obtained as shown in Listing 10:

Listing 10 **SWRL rule for calculating the difference between the internal temperature and the external temperature.**

smarthouse:hasIndoorTemperature(?room, ?internalTemp)∧ smarthouse:hasOutdoorTemperature(?house, ?externalTemp)∧ swrlb:subtract(?internalTemp, ?externalTemp, ?delta)⇒ smarthouse:hasIntExtTempDifference(?room, delta)

Eventually, once an energy wasting situation has been detected, remedial actions could be performed such as warning the tenants or automatically turning off the heating system in the room.

### 3.2. Visual Cueing System Scenario

Largely widespread in the modern domotic landscape, IoT smart light devices are inexpensive colour-changing LED light bulbs remotely controllable. Initially intended for ambient lighting enhancement, smart bulbs can be efficiently used as means to provide visual cues in IoT home automation systems. For example, if tenants are watching the TV or speaking on the phone, to signal the end of the washing machine cycle, the room light could be slightly turned to a blue-like colour instead of relying on an annoying buzzer sound. Most importantly, such a pervasive visual cueing system can potentially result in an efficient low-cost assistive technology in case of hearing impaired tenants [33].

We designed a domotic scenario that exploits the colour-changing features of the IoT house lighting system to signal the presence of a visitor at the entrance door.

The rule in Listing 11 turns the lights colour to red when someone has pressed the doorbell or is standing close to the entrance door. Tenant localisation within the house is performed in order to avoid unnecessary light switching (i.e., only lights in occupied rooms are involved). Alternatively, if indoor tenant localisation is not possible (e.g., due to the lack of presence sensors in every room or it cannot be inferred in another way), a restricted set of light bulbs can be specifically designated for this purpose.

Listing 11 **SWRL rule that signals the presence of a visitor by turning the colour of indoor light to red.**

smarthouse:VisitorAtTheDoor(Entrance)∧ smarthouse:Tenant(?tenant)∧ smarthouse:isLocatedIn(?tenant, ?room)∧ saref:LightSwitch(?light)∧ smarthouse:isLocatedIn(?light, ?room)⇒ smarthouse:TemporaryRedLightColourState(?light)

To detect a visitor at the entrance door, either a presence/motion sensor mounted on the door frame or a smart button can be used. When a presence sensor is used, the rule shown in Listing 12 classifies the entity Entrance into the state VisitorAtTheDoor according to sensor state.

Listing 12 **This SWRL rule is used to detect the presence of a visitor at the door using a volumetric sensor.**

saref:PresenceSensor(P1E)∧ saref:hasState(P1E, OnState)⇒ smarthouse:VisitorAtTheDoor(Entrance)

Similarly, the same operation has to be performed when a doorbell button (i.e., a smart button) is employed. However, since a button is kept pressed only for a few instants of time, a timing mechanism is needed since the entity Entrance should persist in the VisitorAtTheDoor state for a certain number of seconds after the button has been released. This can be achieved by checking the button timestamp, that is, the visitor presence is detected until 10 s have passed since the button press occurred. To retrieve the current time, a virtual IoT device MainClock is used. An example of such a rule is provided in Listing 13.

Listing 13 **SWRL rule to detect the pressure of the doorbell button using the device timestamp.**

smarthouse:hasUnixTimestamp(MainClock, ?clockTime)∧ smarthouse:SmartButton(B1E)∧ smarthouse:hasUnixTimestamp(B1E, ?doorbellTime)∧ swrlb:subtract(?elapsedTime, ?clockTime, ?doorbellTime)∧ swrlb:lessThan(?elapsedTime, $10{,}000$)⇒ smarthouse:VisitorAtTheDoor(Entrance)

The proposed scenario can be easily extended or modified. For example, tenants could receive an alert on their mobile phones if they are not inside and the visual signalling can be automatically disabled during sleep time.

### 3.3. Weather Based Domotic Scenario

IoT weather stations are devices that provide information about the local external environment, including temperature, humidity, wind speed, and rain conditions. In home automation, local climate data can be exploited in several ways, for example, to automatically regulate the curtain position according to natural light and retract it in case of strong wind. One disadvantage of IoT weather stations is that they are expensive equipment, especially compared to other common IoT domotic devices. However, in the SSHS, a physical IoT weather station can be easily replaced by a virtual IoT device that retrieves the same information from an online weather web service.

We designed a domotic scenario that, based on current weather conditions, automatically disables the garden irrigation system in case of rain, thus reducing unnecessary water consumption.

For this scenario, we also implemented a virtual IoT device that acts as a weather station. All the information that the virtual device provides is retrieved from the OpenWeather web service [34]. OpenWeather offers access to current weather data for any location specified by geographic coordinates or city name. Data include information about the weather conditions (e.g., rain, snow, etc.), temperature, humidity, wind speed, and in particular the rain volume for the last 3 h.

Basically, the virtual device consists in a Python script that queries the OpenWeather web service and translates the JSON result into RDF. An excerpt of the output graph is shown in Listing 14.

Listing 14 **An excerpt of the output RDF graph of the weather base station implemented through a virtual IoT device.**

ex:WS1 a smart:SmartWeatherStation ; saref:hasTimestamp ‘‘2020-12-02T14:30:00’’^^xsd:dateTime; smart:hasUnixTimestamp ‘‘1606919400’’^^xsd:integer ; smart:hasSunriseTime 1634018420 ; smart:hasSunsetTime 1634057589 ; smart:hasTemperature 9.29 ; smart:hasWeatherCondition smart:WeatherConditionRain; smart:hasRain3h 3.

We suppose that the irrigation system is activated every day at a fixed time. The rules in Listing 15 prevent the electromechanical valve from opening if the rainfall amount in the last 3 h is greater than 2 mm.

Listing 15 **This SWRL rule prevent the irrigation system to water the garden if the amount of rain in the last 3 h exceeds 2 mm.**

smart:SmartWeatherStation(WS1)∧ smart:hasRain3h:(WS1, ?rainfall)∧ swrlb:greaterThan(?rainfall, 2)⇒ smart:keepClosedState(VALVE1)

Similarly, the irrigation system should be disabled if it has started raining during its functioning. This can be obtained by adding another rule as shown in Listing 16.

Listing 16 **This SWRL rule prevents the irrigation system to water the garden while it is raining.**

smart:SmartWeatherStation(WS1)∧ smart:hasWeatherCondition(WS1, WeatherConditionRain)⇒ smart:keepClosedState(VALVE1)

It has to be noted that two separate rules are necessary since SWRL does not support atom disjunction.

### 3.4. Performance Evaluation

The main advantage of the SSHS over traditional systems based on the trigger-action model is the capability of performing automated reasoning over sensor data and formalised knowledge provided by SWRL rules and OWL ontologies. Knowledge-based systems enabled by DL-OWL reasoners necessitate higher computational capacity compared to systems implemented using general purpose languages such as C++ or Java. Nevertheless, home automation systems are real-time applications that require logic instructions to be executed in a minimal amount of time so as not to cause unwanted delays in automatic actions. Therefore, it is essential that the SSHS completes the inferential process in appropriate time to make it suitable to operate in a real-setting environment.

In order to asses the time performance of the SSHS, similarly to what has been done by Zhai et al. in [35], we executed a set of rules varying the number of devices involved in each experiment repetition, and we measured the average processing time (i.e., the time that the reasoner takes to draw all the inferences) for each configuration. The employed rule-set comprehends: 2 rules that determine whether a room is occupied or unoccupied according to a sensor state (i.e., classification task); 5 rules that classify the air quality into five levels based on the concentration of the CO${}_{2}$ in the room (Wisconsin Department of Health Services. Health effects produced by exposure to CO${}_{2}$. https://www.dhs.wisconsin.gov/chemical/carbondioxide.htm (accessed on 30 March 2022) (i.e., a task that require significant numerical comparisons); 2 rules that make use of the preceding results and an OWL ontology to automatically open the windows in the room in case of high concentration of CO${}_{2}$ if people are present (i.e., a task than involve a chain of inferences and additional OWL axioms). An excerpt of the rule-set is provided in Listing 17.

Listing 17 **An excerpt of the set of rules employed to measure the average processing time.**

smart:CO2Meter(?device)∧ smart:isLocatedIn(?device, ?room)∧ saref:makesMeasurement(?device, ?measurement)∧ saref:hasValue(?measurement, ?value)∧ swrlb:greaterThan(?value, 400)∧ swrlb:lessThanOrEqual(?value, 1000)⇒ smart:CO2GreenLevel(?room) smart:PresenceSensor(?device)∧ saref:hasState(?device, example:OnState)∧ smart:isLocatedIn(?device, ?room)⇒ smart:OccupiedRoom(?room) smart:CO2UnsafeLevel(?room)∧ smart:OccupiedRoom(?room)⇒ smart:WindowsOpen(?room)

The status graph, over which the inferences are drawn, contains the measurements collected by a presence detection sensor and a CO${}_{2}$ meter. Both the sensors are installed in the same room. The presence sensor indicate whether the room is occupied through an OffState-Onstate indication, while the CO${}_{2}$ meter measures the CO${}_{2}$ concentration in the room in ppm. Measurement values and presence states were randomly generated using a Python script. We increased the number of the rooms to vary the number of devices involved in each run of the experiment. Listing 18 provides an example of the status graph generated to test the system time performance.

Listing 18 **An excerpt of the the status graph generated to test the system time performance.**

ex:CO2_1 rdf:type smart:CO2 ; saref:makesMeasurement ex:CO2_1_m1 ; smart:isLocatedIn ex:R1 . ex:CO2_1_m1 rdf:type saref:Measurement;          saref:hasValue ‘‘2186’’^^xsd:float ; saref:isMeasuredIn om:partsPerMillion ; saref:hasTimestamp ‘‘2020-12-02T14:30:00’’^^xsd:dateTime . ex:PRESENCE_1 rdf:type smart:SmartPresence ; saref:hasState ex:OnState ; smart:isLocatedIn ex:R1 .

For each repetition of the experiment we increased the number of devices involved. The inference process has been executed three times for each configuration and the average time calculated. The tests were performed using Protege 5.5.0 on a MacBook Pro equipped with an Intel Core i7 (2.2 GHz, 6-Core) processor and 16 GB (2400 MHz DDR4) of RAM. It is worth to note that existing semantic reasoners are too resource-intensive to be directly ported on resource-constrained devices (such as Raspberry or Arduino) without further engineering efforts. However, promising solutions are currently under investigation [36].

**Figure 4:**
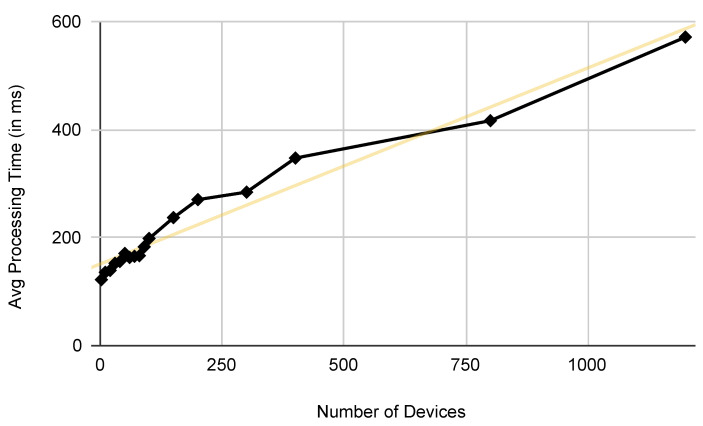
The graph displays the average processing time (in milliseconds) for a number of devices that ranges from 2 to 1200.

Figure 4 shows the results of the experiments. The execution time is specified in milliseconds. As it can be seen from the graph, for a number of devices between 2 and 200 the execution time does not exceed 300 ms which can be considered optimum for real-time operation in a domotic environment where the number of devices of a typical deployment is expected to be in the range of 50 to 100 [37]. This result is significant when compared to the high latency and variability of the popular IFTTT trigger-action system that has been experimentally estimated in the order of seconds [38].

## 4. Discussions

The ubiquity and pervasiveness of the IoT devices in ordinary households offers important opportunities for the home automation sector in many crucial areas such as energy preservation, home safety and living assistance. Notwithstanding, the potential of these devices is still largely under-exploited due to the poor data interoperability and the limited expressivity of the most commonly adopted paradigms for programming domotic scenarios.

The aim of this study was to show that SW technologies can constitute a viable solution not only for tackling the problem of data and device heterogeneity, but also for defining more complex home automation scenarios that can better exploit the potential of IoT technology. To this purpose, we developed a knowledge-based IoT home automation system that can aggregate and combine IoT sensor data with external heterogeneous data sources and analyse them according to formal knowledge expressed by means of OWL ontologies and SWRL rules. In particular, we demonstrated how in such a system the SWRL language can extend and overcome the limitations of the popular trigger-action model by introducing inferential reasoning capabilities directly in domotic scenario definition. The variety of the experiments that we carried out using SAREF as the main reference ontology proved the feasibility of the proposed approach and applicability of it to a standard and well validated context.

Our system opens up to a plethora of possible domotic scenarios that can be implemented ranging from simple actions to more complex automation tasks that make use of common sense knowledge.

Other benefits of the proposed approach include the high degree of explainability of the processes involved. This feature assumes particular importance when home automation is employed to realise in-home healthcare facilities, for example, for elderly care or assisted living. Moreover, due to the high degree of customisation and the overall flexibility, the system could be easily re-adapted for use in other IoT domains where complex automation logic is needed.

However, it is worth to note that at present, even though reuse is partially possible, scenarios require rules to be manually written. Manual rule-writing is not a trivial task but a time consuming and error prone process especially when scenario complexity significantly increases. Therefore, alternative ways to facilitate and potentially automatise the rule creation should be explored. Future research will focus on how to apply machine learning techniques to ease the process of creating rule-based home automation scenarios. Other interesting research directions should address the problems of diagnosis and fault tolerance.

## Abbreviations

The following abbreviations are used in this manuscript:

API	Application Programming Interface
DL	Description Logic
ETSI	European Telecommunications Standards Institute
EUD	End-User Development
IoT	Internet of Things
JSON	JavaScript Object Notation
KGs	Knowledge Graphs
OWL	Web Ontology Language
RDF	Resource Description Framework
SAREF	Smart Applications REFerence (Ontology)
SSHS	Semantic Smart Home System
SW	Semantic Web
SWRL	Semantic Web Rule Language
